# Radiation-induced acute injury of intensity-modulated radiotherapy versus three-dimensional conformal radiotherapy in induction chemotherapy followed by concurrent chemoradiotherapy for locoregionally advanced nasopharyngeal carcinoma: a prospective cohort study

**DOI:** 10.1038/s41598-021-87170-6

**Published:** 2021-04-08

**Authors:** Zexin Yao, Bing Zhang, Jialin Huang, Lei Shi, Biao Cheng

**Affiliations:** 1 School of Public Health, Guangdong Pharmaceutical University, No. 283 Jianghai Avenue, Haizhu District, Guangzhou, 510310 China; 2 School of Pharmaceutical Sciences, Southern Medical University, Guangzhou, 510599 China

**Keywords:** Cancer, Health care, Medical research, Oncology, Risk factors

## Abstract

To address whether the addition of intensity-modulated radiotherapy (IMRT) compared to three-dimensional conformal radiotherapy (3D-CRT) aggravate radiation-induced acute injury of locoregionally advanced nasopharyngeal carcinoma (LANPC) patients with induction chemotherapy (IC) followed by concurrent chemoradiotherapy (CCRT). We conducted a prospective study of 182 patients in the stage III to IVb with biopsy-proven nonmetastatic LANPC who newly underwent radiotherapy and sequentially received IC, followed by CCRT at our institution. Occurring time of radiation-induced toxicities were estimated and compared using the Kaplan–Meier method and Log-rank test. The most severe acute toxicities included oral mucositis in 97.25% and dermatitis in 90.11%. Subset analysis revealed that Grade 3–4 acute dermatitis were significantly higher in the IMRT than 3D-CRT. Oral mucositis and dermatitis were the earliest occurrence of acute injuries (2 years: 60.44% and 17.58%). Patients in IMRT group achieved significantly lower risk of bone marrow toxicity, but higher risk of leukopenia and gastrointestinal injury. Multivariate analyses also demonstrated that IMRT, female gender and hepatitis were the independent prognostic factors for bone marrow toxicity. In a combined regimen of IC followed by CCRT for the treatment of LANPC, IMRT seems to be an aggressive technique with a trend towards increased gastrointestinal and hematological toxicities, but decreased bone marrow toxicity than those treated with 3D-CRT. This study provides a comprehensive summary of prospective evidence reporting the side effects in the management of LANPC patients. We quantify the occurrence risks of chemoradiotherapy-induced acute injuries through analysis of time-to-event.

## Introduction

Nasopharyngeal carcinoma (NPC) is a special cancer of head and neck, with a high incidence (15 to 50 cases per 100,000 persons annually) and main causes of cancer death (50,000 persons per year) in endemic regions such as Southern China, Southern Asia, the Middle East, Northern Africa, Singapore, and Malaysia^1^. Radiotherapy (RT) is used in the primary treatment for NPC because of its inherent anatomic site and high radiosensitivity^2^. The currently recommended standard regimen is the addition of concurrent chemotherapy (CCRT) for NPC patients who receive RT with or without adjuvant chemotherapy being superior to radiotherapy alone^3^. Nevertheless, induction chemotherapy (IC) combined with the established CCRT regimen has attracted attention for the management of locoregionally advanced NPC (LANPC) during recent decades^4^. The use of IC followed by definitive CCRT was associated with improved clinical outcomes, which could decrease distant metastases. In the National Comprehensive Cancer Network (NCCN) clinical practice guideline for patients with the stage III to IVb NPC, the preferred recommendation is participating in clinical trials, while the category 2A and 2B recommendations are, respectively, IC followed by CCRT and CCRT alone. IC consistently results in higher response and exerts a pronounced effect on distant metastases. However, the treatment inevitably carries higher rates of acute toxicities and late complications to normal tissues, including dermatitis, oral mucositis, bone marrow and gastrointestinal toxicity with a significant impact on the quality of life, unplanned treatment interruptions and leading to the development of tumors^5^. It is a great challenge to provide accurate radiative dose with minimal irradiation adverse events^6,7^.

More recently, the selectivity of radiation techniques has improved tremendously first by the replacement of two-dimensional (2D) planning by three-dimensional conformal radiotherapy (3D-CRT) techniques with a low rate of acute treatment toxicities and excellent rates of short-term local control. 3D-CRT is an advanced treatment technique that can irradiate the target area through multiple fields (usually 5–7 fields) to align the tumor with the radiation target through three-dimensional directions. It makes the target dose distribution more reasonable and reduces exposure to adjacent normal tissue^8^. Besides, intensity-modulated radiation therapy (IMRT) is an advanced form of radiation technique allowing a more target coverage and dose homogeneity that delivers higher dose to the tumor and conforms to the scattering irradiation volume to critical organs while keeping less toxicity to skin and surrounding tissues^9,10^. Multiple dosimetry studies have demonstrated that IMRT has a similar survival and low risk on the occurrence of acute adverse events over 3D-CRT in the management of breast, head and neck cancer^11,12^. To the best of our knowledge, more studies mainly focused on the local control and survival of tumors, but few compared 3D-CRT with IMRT for acute skin reaction and other side effects of LANPC irradiation with a combined strategy of IC-CCRT^13^. However, data to comprehensive IMRT with IC-CCRT in control arm was limited, the IMRT has not been shown to be superior to 3D-CRT in acute toxicity.

Therefore, a prospective cohort study with the Kaplan–Meier method and Log-rank analysis was performed to compare IMRT with 3D-CRT combined induction with concurrent chemotherapy for evaluating radiation-induced toxicities in a large patient collective on LANPC. The time of occurrence for radiation-induced acute injuries is brought forward which is also one of creation in research perspective. In this paper, we reported the treatment outcome.

## Methods and materials

### Design

We conducted a prospective cohort study for LANPC patients at the department of oncology, General Hospital of Southern Theater Command in Guangzhou, China, from January 2016 to December 2018. Those with diagnosis was confirmed by histopathology were enrolled if they had stage III–IVb NPC, as defined according to the 7th edition of the American Joint Committee on Cancer (AJCC) stage criteria. Inclusion criteria were aged ≥ 18 years with a completion of radical radiotherapy and patients with weekly on-treatment visit documentation of acute treatment related toxicities. Patients with tumor treated with induction chemotherapy prior to concurrent chemoradiation were included in the present analysis. A patient would be excluded if he or she had: (a) a history of radiotherapy to the head or neck; (b) Karnofsky performance score (KPS) < 70; (c) severe heart, lung, liver or kidney dysfunctions unsuitable for radiotherapy; (d) transfer to other hospitals for treatment of irradiation; (f) their NPC was in stage I or II. The medical follow-up included medical history with physical examination, adverse events, hematologic and chemical tests, panendoscopy with biopsies and radiologic evaluation. A patient's stage was determined based on the Union for International Cancer Control/American Joint Cancer Committee (UICC/AJCC) TNM classification. Tumor histology was classified according to the World Health Organization classification. The protocol was approved by the Ethics Committee at General Hospital of Southern Theater Command, and the study was conducted in accordance with the principles of the Declaration of Helsinki. Written informed consent was obtained from all patients included in the study and the data was anonymized and maintained with confidentiality. Also the data obtained from the medical records without research-related intervention, the Clinical trial registration was not applicable.

### Radiotherapy

The planning CT dataset was acquired using a 16-detector scanner. All patients underwent RT with 6 to 10 MV photon linear accelerators using either 3D-CRT or IMRT. Helical TomoTherapy combines a rotational IMRT with a translational movement of the couch. CT image data were reconstructed as 5-mm sections for 3D-CRT and and IMRT. The treatment using radiotherapy technologies and fractionated dose with an individually optimized plan for each patient was considered mainly according to the wishes of the patients. The gross tumor volume (GTV) was defined by the International Commission on Radiation Units and Measurements Reports 50 and 62, including gross primary tumors (GTVnx) and involved lymph nodes (GTVnd) determined from clinical and imaging examinations. The clinical target volume (CTV) for high-risk (CTV1) should include the entire nasopharynx, retropharyngeal lymph nodal regions and any high-risk nodal regions. The CTV for low-risk (CTV2) was recognized as the next station of the positive lymph nodes and the elective neck area. The GTVnx plus a 3 mm margin was defined as planning gross tumor volume (PGTVnx) with 60–70 Gy and the GTVnd plus a 5 mm margin was defined as PGTVnd with 60–70 Gy. Other two planning target volumes (PTV1 and PTV2) were defined as CTV1 plus a 3 mm margin with 60 Gy and CTV2 plus a 3 mm margin with 54 Gy. Both patient groups were treated with one fraction of 2.00–2.80 Gy daily, five times per week.

### Chemotherapy

All patients were received one to four cycles of platinum-based IC before radiotherapy for LANPC and one to three cycles CCRT with platinum-based treatment (40 mg/m^2^) for 3 days. The most common induction regimens mainly included TPF (Taxol 210 mg/m^2^/day on day 1, platinum 40 mg/m^2^/day on days 1–3, and 5-fluorouracil [5-FU] 750 mg/m^2^/day on days 1–3), TP (Taxol 210 mg/m^2^/day on day 1, platinum 40 mg/m^2^/day on days 1–3), PF (platinum 40 mg/m^2^/day on days 1–3, 5-FU 750 mg/m^2^/day on days 1–3). The treatment plan was administered at the discretion of the treating physicians.

### Follow up and toxicity evaluation

All the subjects underwent weekly examinations for acute toxicities and treatment response during radiation therapy. The evaluated radiation-induced injuries including oral mucositis, skin injury, dermatitis, hyperpigmentation, bone marrow toxicity, leukopenia, anemia and gastrointestinal toxicity. They were recorded the date prospectively when it first appeared and scored according to the Radiation Therapy Oncology Group (RTOG). The index date was the start date for RT and the date of toxicity evaluation in the cohort. If a patient experienced RTOG grade 4 toxic effects or was hospitalized, treatment was either delayed or suspended. The primary endpoint was development of RTOG grade with radiation-induced acute injuries. Patients were followed up every 3 months for 2 years after treatment.

### Treatment effect

The short-term curative effects of the two groups of patients were evaluated three months after radiotherapy. The local control rate of treatment includes rate of complete remission (CR) and partial relief (PR). Secondary outcomes of this study included overall survival (OS, calculated as the time from start of treatment until death from any cause), locoregional progression-free survival (LRFS, defined as the time from start of treatment until recurrence in the nasopharyngeal or neck area), distant metastasis-free survival (DMFS, defined as the time from start of treatment until detection of distant metastasis) and progression-free survival (PFS, defined as the time from start of treatment until disease progression). Each follow-up included physical examinations, nasoendoscopy, chest imaging, ultrasound of abdomen, MRI scan of the nasopharynx and bone scan. Additional examinations were performed when it is indicated to confirm local recurrence or distant metastasis by an experienced doctor. Patients without the information on disease progression or lost to follow-up were treated as censored data.

### Statistical analysis

Patterns of time-to-event were analyzed: the incidence of periods and the median days of occurred acute toxicities after IMRT or 3D-CRT were estimated using the Kaplan–Meier methods, compared with the Log-rank test. Baseline characteristics including age, gender, cigarette smoking, alcohol consumption, AJCC clinical stage, TNM category, comorbidity, irradiation fraction dose, BMI and chemotherapy regimens, differences in proportions between IMRT and 3D-CRT groups were assessed using Chi-square (*χ2*) test or Fisher’s exact test, and the quantitative variation such as age, BMI, irradiation fraction dose and radiotherapy dose were compared by means of Student’s *t* test or Kruskal-Willis test where appropriate. Cox proportional hazard model was used to identify potentially independent prognostic factors, and the proportional hazards assumption was tested with Schoenfeld residuals. The hazard ratio (HR) and its 95% confidence interval (95% CI) were used to indicate the prognostic value of risk factors. The Kaplan–Meier method was used to estimate OS, LRFS, PFS, DMFS. We further performed interaction analysis to explore the variation of treatment effect in subgroups, including clinical staging. A *P*-value of < 0.05 from two-sided tests was defined as being of statistically significant. All statistical analyses were performed with the SAS 9.4 statistical software (SAS Institute Inc. USA, https://www.sas.com).

## Results

### Patients and treatments characteristics

**Figure 1:**
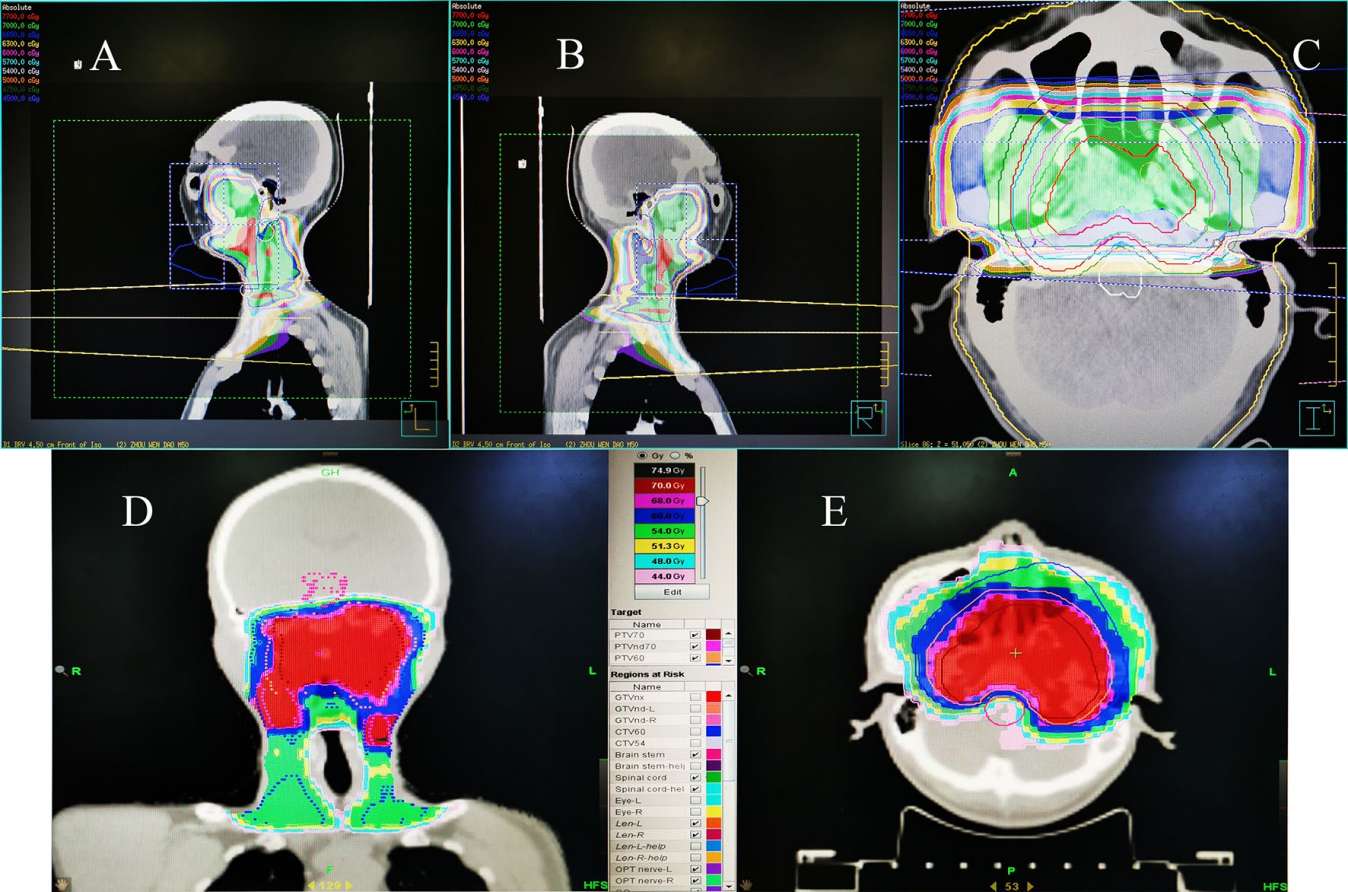
The dose distribution diagram of neck and nasopharyngeal for 3D-CRT (**A**–**C**) and IMRT (**D**, **E**).

**Figure 2:**
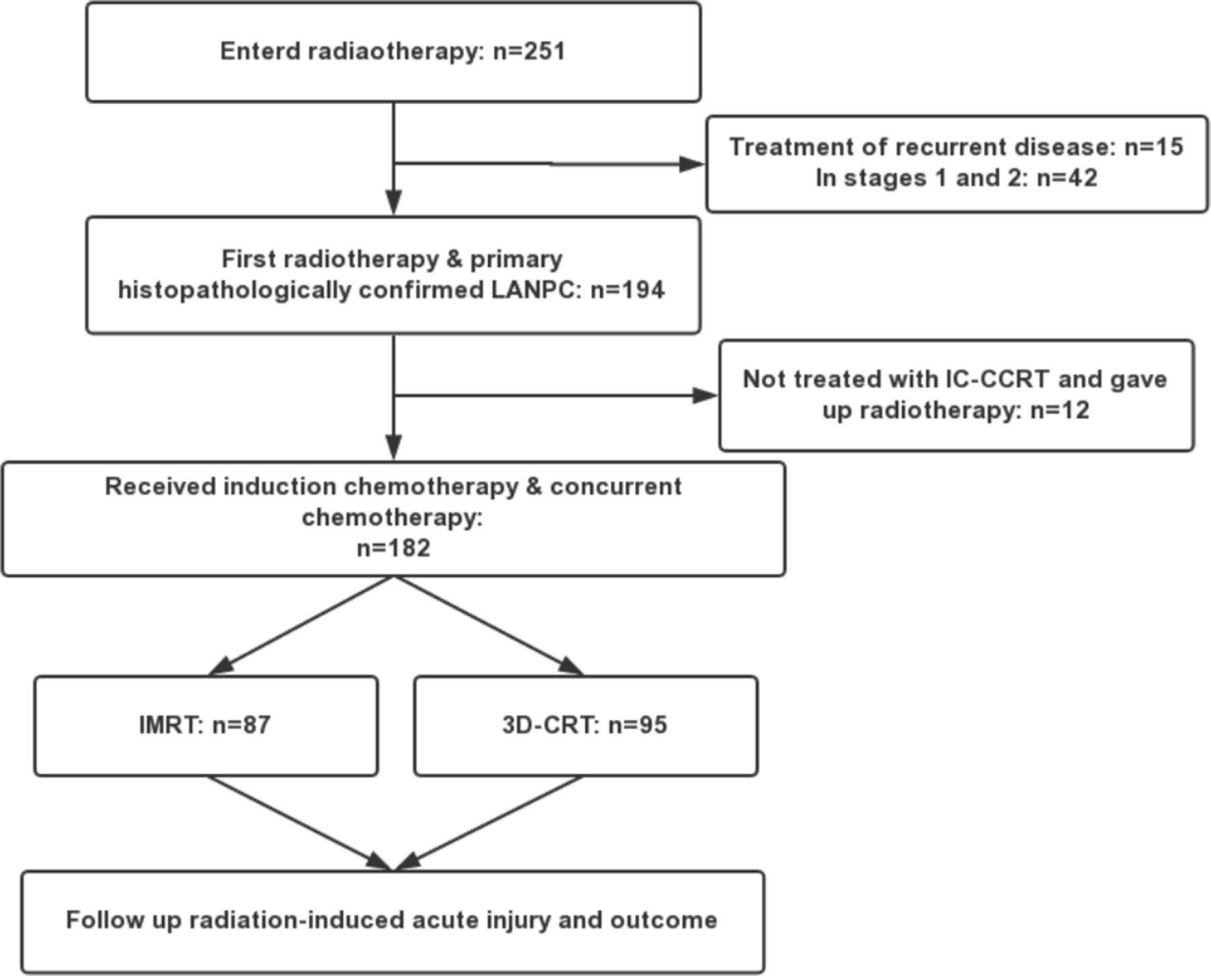
The consolidated standards of reporting trials (CONSORT) flow-chart for the study.

The dose distribution diagram of neck and nasopharyngeal for 3D-CRT and IMRT have been attached in Fig. 1. Both injuries were located in the right pons within the high-dose irradiated field, and were considered radiation-induced. The consolidated standards of reporting trials (CONSORT) flow-chart for the study is depicted in Fig. 2. The median follow-up time was 24 months (range 1–46 months) for the entire cohort of patients. Table 1 summarizes the baseline data and treatment characteristics of 182 patients for IMRT and 3D-CRT groups included in the trial. A total of 87 patients (mean age: 49.05 ± 11.52 years, range 23–76) received IMRT and 95 (mean age: 51.22 ± 11.26 years, range 27–78) (*P* = 0.4767) received conventional 3D-CRT. In both groups, there were more males than females with a male: female ratio of approximately 4.1:1. The most patients presented with stage III (47.25%), stage IVa (44.51%) and stage IVb (8.24%) disease. There were 77.01% patients with T3-4 in IMRT group and 62.11% in 3D-CRT group (*P* = 0.0573), 74.71% vs. 82.11% with N2-3 (*P* = 0.1725), overall stage was balanced (*P* = 0.8323). The median radiotherapy dose was 68.68 Gy in both groups and the daily dose was always 219 cGy in IMRT group and 202 cGy in 3D-CRT group. Furthermore, TPF of induction chemotherapy and nedaplatin of concurrent chemotherapy were applied in a higher fraction of individuals receiving IMRT (IMRT vs. 3D-CRT: 26.44% vs. 11.58%, *P* = 0.0015; 36.78% vs. 9.47%, *P* < 0.0001). There were no significant differences in age, gender, current smoker and drinker, tumor TNM stage, clinical stage, comorbidities, process of cancer, BMI, total radiation dosage, length of RT to CC and IC to RT. In multiple Logistic regression analysis, there is no bearing with the choices of two radiotherapy technologies. Overall, these characteristics were largely representative of nasopharyngeal cancer population in the community, ensuring good external validity with widespread applicability and generalization of results.

**Table 1:** Demographics and clinical characteristics.

Characteristics	All (n = 182)	IMRT (n = 87)	3D-CRT (n = 95)	*P* value
	N (%)	N (%)	N (%)	
**Gender**				
Male	146 (80.22)	66 (75.86)	80 (84.21)	0.1579
Female	36 (19.78)	21 (24.14)	15 (15.79)	
**Age (years)**				
≤ 60	31 (17.03)	16 (18.39)	15 (15.79)	0.6410
> 60	151 (82.97)	71 (81.61)	80 (84.21)	
Current smoker	43 (23.63)	17 (19.54)	26 (27.37)	0.2143
Current drinker	33 (18.13)	13 (14.94)	20 (21.05)	0.2852
**BMI**				
Underweight	87 (47.8)	42 (48.28)	45 (47.37)	0.0909
Normal	45 (24.73)	16 (18.39)	29 (30.53)	
Overweight	50 (27.47)	29 (33.33)	21 (22.11)	
Comorbidity	46 (25.27)	21 (24.14)	25 (26.32)	0.7356
**TNM category**				
T1–2	55 (30.22)	20 (22.99)	35 (36.84)	0.0573
T3–4	126 (69.23)	67 (77.01)	59 (62.11)	
N0–1	38 (20.88)	22 (25.29)	16 (16.84)	0.1725
N2–3	143 (78.57)	65 (74.71)	78 (82.11)	
M0	154 (84.62)	74 (85.06)	80 (84.21)	0.6960
M1	15 (8.24)	8 (9.20)	7 (7.37)	
**Clinical stage**				
III	86 (47.25)	42 (48.28)	44 (46.32)	0.8323
IVa	81 (44.51)	37 (42.53)	44 (46.32)	
IVb	15 (8.24)	8 (9.20)	7 (7.37)	
**RT fraction**				
Conventional	82 (45.05)	1 (1.15)	81 (85.26)	** < 0.0001**
Hypo-fractionated	74 (40.66)	70 (80.46)	4 (4.21)	
**IC regimens drugs**				
TP	123 (67.58)	45 (51.72)	78 (82.11)	**0.0015**
TPF	34 (18.68)	23 (26.44)	11 (11.58)	
PF	2 (1.10)	2 (2.30)	0 (0)	
**CC platinum-based drugs**				
Cisplatin	98 (53.85)	41 (47.13)	57 (60.00)	** < 0.0001**
Nedaplatin	41 (22.53)	32 (36.78)	9 (9.47)	
**Short-term effects**				
CR + PR	91 (50.00)	57 (65.52)	34 (35.79)	**0.0002**
SD + PD	87 (47.80)	30 (34.48)	57 (60.00)	

*IMRT* intensity modulated radiation therapy, *3D-CRT* three-dimensional conventional radiotherapy, *RT* radiotherapy, *IC* induction chemotherapy, *CC* concurrent chemotherapy, *CR* complete remission, *PR* complete remission.

*P* values were calculated with Chi-square (χ2) test or Fisher’s exact test. The use of bold formatting means *P* < 0.05.

### Acute side effects of treatment

Relevant side effects were reported by most patients. Acute complications are reported in Table 2. At the end of follow-up, almost all patients described at least some kind of radiation-induced injuries (98.85% in IMRT group and 97.89% in 3D-CRT, *P* = 1.0000). Acute dermatitis was observed 89.66% of patients in IMRT and 90.53% in 3D-CRT group (*P* = 0.8441). Oral mucositis occurred 95.40% and 98.95% of patients in IMRT and 3D-CRT groups, respectively (*P* = 0.1946). Bone marrow toxicity was present 22.99% of patients treated in IMRT group and 53.68% in 3D-CRT group (*P* < 0.0001). Weight loss was present 67.82% of patients treated in IMRT group and 70.53% in 3D-CRT group (*P* = 0.1656). There was a significantly better control of hyperpigmentation, bone marrow toxicity and otitis media in IMRT, but a trend toward better moist desquamation, erythema, xerostomia and gastrointestinal injury in patients treated with 3D-CRT remains. Dermatitis, exudate, oral mucositis, anemia, leukopenia and so on were no differently observed at IMRT and 3D-CRT. The proportions of patients with physician-rated RTOG grade 3 or worse acute dermatitis, oral mucositis and bone marrow toxicity were 40.11%, 58.79% and 17.58%. Severe acute dermatitis was significantly higher in IMRT arm as compared RTOG I-II (*P* = 0.0482), but oral mucositis and bone marrow toxicity were not significantly lower.

**Table 2:** Incidence of radiation-injuries by radiotherapy modality.

Variables	All (n = 182)	IMRT (n = 87)	3D-CRT (n = 95)	*P* value
	N (%)	N (%)	N (%)	
Radiation-induced injury	179 (98.35)	86 (98.85)	93 (97.89)	1.0000
Dermatitis	164 (90.11)	78 (89.66)	86 (90.53)	0.8441
RTOG I–II	91 (50.00)	37 (42.53)	54 (56.84)	**0.0482**
RTOG III–IV	73 (40.11)	41 (47.13)	32 (33.68)	
Flush	57 (31.32)	32 (36.78)	25 (26.32)	0.1283
Hyperpigmentation	112 (61.54)	45 (51.72)	67 (70.53)	**0.0092**
Dry desquamation	69 (37.91)	30 (34.48)	39 (41.05)	0.3615
Moist desquamation	24 (13.19)	20 (22.99)	4 (4.21)	**0.0002**
Erythema	12 (6.59)	12 (13.79)	0 (0)	**0.0002**
Moderate edema	9 (4.95)	8 (9.20)	1 (1.05)	**0.0147**
Ulceration	64 (35.16)	29 (33.33)	35 (36.84)	0.6205
Exudate	4 (2.20)	1 (1.15)	3 (3.16)	0.6223
Infection	7 (3.85)	5 (5.75)	2 (2.11)	0.2616
Pain	31 (17.03)	18 (20.69)	13 (13.68)	0.2092
Pruritus	22 (12.09)	16 (18.39)	6 (6.32)	**0.0126**
Oral mucositis	177 (97.25)	83 (95.4)	94 (98.95)	0.1946
RTOG I–II	70 (38.46)	30 (34.48)	40 (42.11)	0.3842
RTOG III–IV	107 (58.79)	53 (60.92)	54 (56.84)	
Hyperaemia	88 (48.35)	36 (41.38)	52 (54.74)	0.0717
Ulceration	124 (68.13)	56 (64.37)	68 (71.58)	0.2970
Edema	12 (6.59)	6 (6.90)	6 (6.32)	0.8747
Flush	44 (24.18)	21 (24.14)	23 (24.21)	0.9909
Leukasmus	67 (36.81)	31 (35.63)	36 (37.89)	0.7519
Xerostomia	71 (39.01)	42 (48.28)	29 (30.53)	**0.0142**
Weight loss	126 (69.23)	59 (67.82)	67 (70.53)	0.1656
< = 5%	13 (7.14)	5 (5.75)	8 (8.42)	0.5234
> 5%	113 (62.09)	54 (62.07)	59 (62.11)	
Bone marrow toxicity	71 (39.01)	20 (22.99)	51 (53.68)	** < 0.0001**
RTOG I–II	34 (18.68)	10 (11.49)	24 (25.26)	0.6875
RTOG III–IV	32 (17.58)	8 (9.20)	24 (25.26)	
Gastrointestinal injury	37 (20.33)	25 (28.74)	12 (12.63)	**0.0070**
Nausea and vomiting	69 (37.91)	32 (36.78)	37 (38.95)	0.7636
Anemia	27 (14.84)	11 (12.64)	16 (16.84)	0.4261
Leukopenia	71 (39.01)	37 (42.53)	34 (35.79)	0.3518
Pneumonitis	5 (2.75)	4 (4.60)	1 (1.05)	0.1946
Encephalopathy	7 (3.85)	2 (2.30)	5 (5.26)	0.4472
Otitis media	10 (5.49)	1 (1.15)	9 (9.47)	**0.0193**

*IMRT* intensity modulated radiation therapy, *3D-CRT* three-dimensional conventional radiotherapy, *RTOG* Radiation Therapy Oncology Group.

*P* values were calculated with Chi-square (χ2) test or Fisher’s exact test. The use of bold formatting means *P* < 0.05.

**Figure 3:**
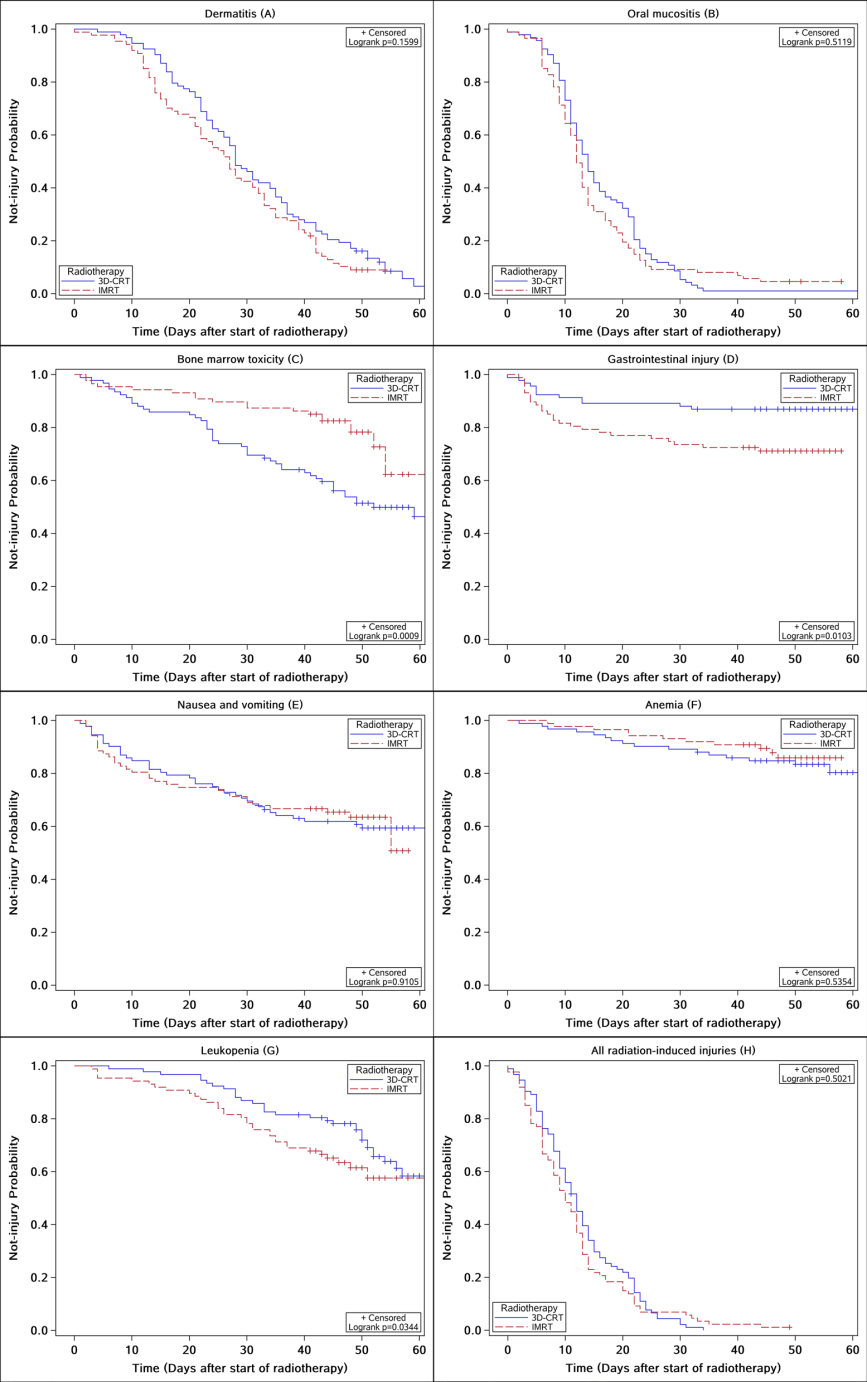
Kaplan–Meier curves for dermatitis (**A**), oral mucositis (**B**), bone marrow toxicity (**C**), gastrointestinal injury (**D**), nausea and vomiting (**E**), anemia (**F**), leukopenia (**G**) and all radiation-induced injuries (**H**) in NPC patients treated with IMRT versus 3D-CRT.

Figure 3 show the radiation-induced toxicity curves for patients receiving IMRT vs. 3D-CRT regimens of radiotherapy. The time of occurrence for radiation-induced acute injuries was performed in Table 3 and oral mucositis occurred earliest. Median times to occur oral mucositis was 12 days in IMRT group and 13 days in 3D-CRT group. Next, acute dermatitis was 26 days in IMRT group and 28 days in 3D-CRT group. For the whole study population, 2-weeks morbidity for dermatitis, oral mucositis, bone marrow and gastrointestinal toxicities were 24.14%, 66.67%, 5.75%, 20.69% in IMRT group and 11.58%, 54.74%, 16.84%, 13.68% in 3D-CRT group, respectively. The 4-weeks incidence were 56.32% vs. 52.63%, 90.80% vs. 89.47%, 10.34% vs. 28.42% and 25.29% vs. 13.68%, respectively. Comparison of toxicities demonstrated that IMRT was associated with lower bone marrow toxicity than 3D-CRT (HR = 0.426, *P* = 0.0009), but higher gastrointestinal injury (HR = 2.383, *P* = 0.0103) and leukopenia (HR = 1.444, *P* = 0.0344). There were no significant differences in other acute toxicities between 3D-CRT and IMRT in univariate survival analysis.

**Table 3:** Kaplan–Meier method compared for radiation-induced injuries between IMRT and 3D-CRT [N (%)].

Acute toxicity	All (n = 182)			IMRT (n = 87)			3D-CRT (n = 95)			HR (95% Cl)
	2 weeks	4 weeks	6 weeks	2 weeks	4 weeks	6 weeks	2 weeks	4 weeks	6 weeks	
Radiation-induced injury	131 (71.98)	172 (94.51)	180 (98.90)	67 (77.01)	81 (93.10)	85 (97.70)	64 (67.37)	91 (95.79)	95 (100)	1.102 (0.818–1.485)
Dermatitis	32 (17.58)	99 (54.40)	148 (81.32)	21 (24.14)	49 (56.32)	75 (86.21)	11 (11.58)	50 (52.63)	73 (76.84)	1.243 (0.910–1.698)
Oral mucositis	110 (60.44)	164 (90.11)	176 (96.70)	58 (66.67)	79 (90.80)	82 (94.25)	52 (54.74)	85 (89.47)	94 (98.95)	1.101 (0.815–1.487)
Bone marrow toxicity	21 (11.54)	36 (19.78)	61 (33.52)	5 (5.75)	9 (10.34)	20 (22.99)	16 (16.84)	27 (28.42)	41 (43.16)	0.426 (0.252–0.720)*
Gastrointestinal injury	31 (17.03)	35 (19.23)	45 (24.73)	18 (20.69)	22 (25.29)	28 (32.18)	13 (13.68)	13 (13.68)	17 (17.89)	2.383 (1.197–4.745)*
Nausea and vomiting	40 (21.98)	54 (29.67)	72 (39.56)	20 (22.99)	25 (28.74)	32 (36.78)	20 (21.05)	29 (30.53)	40 (42.11)	0.973 (0.605–1.565)
Anemia	9 (4.95)	19 (10.44)	33 (18.13)	2 (2.30)	6 (6.90)	14 (16.09)	7 (7.37)	13 (13.68)	19 (20.00)	0.783 (0.360–1.701)
Leukopenia	12 (6.59)	30 (16.48)	55 (30.22)	7 (8.05)	16 (18.39)	33 (37.93)	5 (5.26)	14 (14.74)	22 (23.16)	1.444 (1.889–2.348)*

*IMRT* intensity modulated radiation therapy, *3D-CRT* three-dimensional conventional radiotherapy.

Hazard ratios (HR) were calculated with the Cox proportional hazards model, *P* values were calculated with the log-rank test, *means *P* < 0.05.

**Table 4:** Prediction of radiation-induced acute injuries in Cox proportional hazards analysis.

Characteristics	Bone marrow toxicity		Gastrointestinal injury		Leukopenia	
	HR (95% Cl)	*P* value	HR (95% Cl)	*P* value	HR (95% Cl)	*P* value
3D-CRT versus IMRT	0.429 (0.209–0.881)	0.0211	1.885 (0.908–3.915)	0.0889	1.360 (1.244–1.973)	0.0452
Gender (male vs. female)	2.07 (0.99–4.327)	0.0531				
**Comorbidity**						
Hepatitis	2.152 (0.983–4.713)	0.0552				
M category (M0 vs. M1)					2.215 (0.781–6.28)	0.1349

Characteristics	Oral mucositis		Dermatitis			
	HR (95% Cl)	*P* value	HR (95% Cl)	*P* value		
Gender (male vs. female)	1.529 (0.927–2.522)	0.0963				
**Comorbidity**						
Hepatitis	2.136 (1.145–3.987)	0.0171				
IC regimens drugs (TPF vs.TP)	0.548 (0.335–0.896)	0.0165				
IC regimens drugs (TPF vs. PF)	0.203 (0.027–1.529)	0.1217				
RT fraction (conventional vs. hypo-fractionated)			1.947 (1.225–3.096)	0.0049		
CC platinum-based drugs (cisplatin vs. nedaplatin)			0.626 (0.372–1.054)	0.0782		

*IMRT* intensity modulated radiation therapy, *3D-CRT* three-dimensional conventional radiotherapy, *IC* induction chemotherapy, *CC* concurrent chemotherapy, *RT* radiotherapy.

Hazard ratios (HR) were calculated with the Cox proportional hazards model, *P* values were calculated with the log-rank test.

### Predictive factors

A Cox proportional hazards analysis was showed in Table 4, 3D-CRT was significantly associated with the bone marrow toxicity in our survival analysis (Hazard ratio [HR] = 0.429, *P* = 0.0211). And IMRT was significantly related to gastrointestinal toxicity (HR = 1.885, *P* = 0.0889) and leukopenia (HR = 1.360, *P* = 0.0452). Only female gender (HR = 2.070, *P* = 0.0531) and hepatitis (HR = 2.152, *P* = 0.0552) were significantly associated with an unfavorable bone marrow toxicity. If the analysis was restricted to leukopenia, M1 of TNM category were detected as the additional predictors (HR = 2.215, *P* = 0.1349). The technologies of radiotherapy could be an independent factor of gastrointestinal injury. It was demonstrated that oral mucositis was not associated with IMRT, which related to female, hepatitis and TPF of IC regimens drugs. Hypo-fractionated radiotherapy and cisplatin were detected as the exclusively predictive factors of dermatitis.

### Outcome and efficacy of treatment

**Figure 4:**
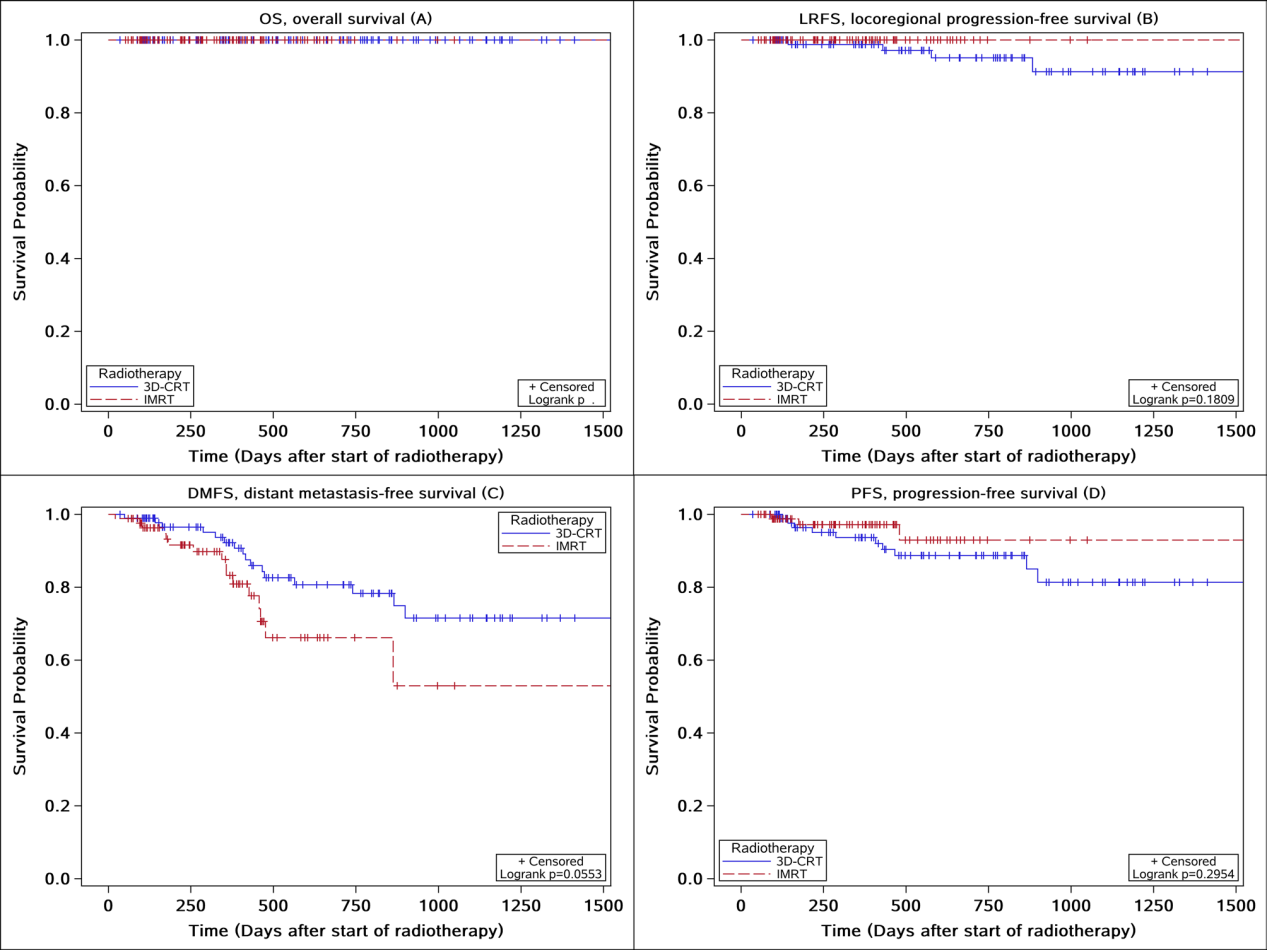
The survival curves of overall survival (OS) (**A**), locoregional progression-free survival (LRFS) (**B**), distant metastasis-free survival (DMFS) (**C**) and progression-free survival (PFS) (**D**) for NPC patients received IMRT versus 3D-CRT.

For treatment outcome, Fig. 4 shows the survival curves that there were no differences in mortality, recurrence-free and distant metastasis and progression-free at 2 years after treatment in patients treated with IMRT and 3D-CRT. The 2-year Kaplan–Meier estimates of OS, LRFS, DMFS and PFS were 100% vs. 100% (*P* = 1.0000), 100.00% vs. 95.79% (*P* = 0.1809), 81.61% vs. 83.16% (*P* = 0.0553) and 96.55% vs. 89.47% (*P* = 0.2954) for the IMRT and 3D-CRT arms respectively. The results of subgroup analysis of survival showed in Table 5, there was no statistically significant difference in OS, LRFS, DMFS and PFS between the IMRT group and the 3D-CRT group in each clinical stage (*P* > 0.05). At 3 months after the end of treatment, patients treated with IMRT and 3D-CRT had reported a local control rate rates were higher in patients receiving IMRT compared to patients with 3D-CRT (65.52% vs. 35.79%, *P* = 0.0002). In multivariate regression analysis, the outcome and efficacy of treatment were not significantly associated with two groups. There was a risk factor of anemia for short-term efficacy (HR = 3.681, 95% *Cl*: 1.117–12.129).

**Table 5:** Kaplan–Meier method compared for survival rates between IMRT and 3D-CRT.

Survival rates	All	IMRT	3D-CRT	HR (95% Cl)	*P* value
	N (%)	N (%)	N (%)		
OS	182 (100)	87 (100)	95 (100)	–	1.0000
III	86 (100)	42 (100)	44 (100)	–	1.0000
IVa	81 (100)	37 (100)	44 (100)	–	1.0000
IVb	15 (100)	8 (100)	7 (100)	–	1.0000
LRFS	178 (97.80)	87 (100)	91 (95.79)	–	0.1809
III	85 (98.84)	42 (100)	43 (97.73)	–	0.6949
IVa	78 (96.30)	37 (100)	41 (93.18)	–	0.1447
IVb	15 (100)	8 (100)	7 (100)	–	1.0000
DMFS	150 (82.42)	71 (81.61)	79 (83.16)	1.986 (0.973–4.056)	0.0553
III	74 (86.05)	37 (88.10)	37 (84.09)	1.565 (0.475–5.162)	0.4585
IVa	66 (81.48)	30 (81.08)	36 (81.82)	1.668 (0.585–4.756)	0.3334
IVb	10 (66.67)	4 (50.00)	6 (85.71)	7.15 (0.757–67.531)	0.0502
PFS	169 (92.86)	84 (96.55)	85 (89.47)	0.503 (0.136–1.866)	0.2954
III	83 (96.51)	42 (100)	41 (93.18)	–	0.1808
IVa	75 (92.59)	36 (97.30)	39 (88.64)	0.358 (0.041–3.155)	0.3353
IVb	11 (73.33)	6 (75.00)	5 (71.43)	0.994 (0.138–7.159)	0.9952

*IMRT* intensity modulated radiation therapy, *3D-CRT* three-dimensional conventional radiotherapy, *OS* overall survival, *LRFS* locoregional relapse-free survival, *DMFS* distant metastasis-free survival, *PFS* progression-free survival.

Hazard ratios (HR) were calculated with the Cox proportional hazards model, *P* values were calculated with the log-rank test.

## Discussion

The superiority of IC-CCRT combined with radiation in patients was first demonstrated in the landmark which replaced chemoradiotherapy alone with induction platinum-based chemotherapy followed by concurrent high dose cisplatin with radiotherapy^14^. The scheme of induction therapy can effectively alleviate the lesions and create improved radiotherapy conditions for LANPC, especially in patients with giant lesions, achieving better survival and prognosis. However, one of the outcomes of induction chemotherapy can increase radiation-induced toxicities and adverse events, such as dermatitis or oral mucositis^9,15,16^. There is currently no well-defined standard of care in the management of patients with radiation-induced acute injuries. Radiation-related injury, which frequently occurred in patients treated with NPC, could cause physical and mental suffering due to pain, ulcer, exudate, various cosmetic problem and reduced quality of life^17,18^. To reduce acute toxicity, a better focusing of radiation were actively studied and avoiding the integument. IMRT and 3D-CRT have been demonstrated an improved treatment outcome and lower skin toxicity in other head and neck cancer, they are the primary radiotherapy techniques for cancer patients during recent decades^19^. However, the prevalence of radiation-related complication with induction chemoradiotherapy in the current literature varies widely^20–22^. There is still a lack of evidence and controversy comparing IMRT with 3D-CRT for LANPC. To the best of our knowledge, there is no randomized controlled comparison for IMRT vs. 3D-CRT in LANPC treated with IC-CCRT bringing about acute toxicity. For this reason, we should pay attention to injury-sparing IMRT and 3D-CRT, which is an active method to reduce radiation-related acute toxicity. The investigation of the occurred timing for acute adverse events will be benefit to prevent these radiation-related toxicities during the treatment^23^. The purpose of this study was to provide a comprehensive description of radiation-induced toxicity rates and acute side effects for IMRT vs. 3D-CRT in LANPC with platinum-based IC-CCRT.

Notably, the present study is the first to prospectively measure and report the occurred time of acute toxicity after initiation of radiation treatment and comparison for two irradiation techniques within the analysis of a Cox proportional hazards model. That provided a new method for further study between radiation injury and radiotherapy in cancer patients after then. Our novel finding of a differential effect between the toxic risk of 2–6 weeks and the proportion of acute injury on the treatment compared with 3D-CRT that IMRT may confer a substantive disadvantage to patients been put off treatment plan for bringing forward adverse events. A Cox proportional hazards model of this study stated less bone marrow toxicity and worse gastrointestinal control, a trend toward earlier occurred white blood cell toxicity in patients treated with IMRT.

Acute skin toxicity in the form of radiation dermatitis or skin hyperpigmentation is a common problem experienced by patients undergoing NPC irradiation. The most common acute adverse events were oral mucositis and dermatitis, which occurred in 96.43% and 88.84% of the patients, the earliest occurrence of oral mucositis was observed during the treatment. A possible explanation for these results may come from susceptible to external infection and close-range irradiation. A phase III randomized trial of IC-CCRT in patients with locally advanced head and neck cancer stated that the most common toxicities were mucositis and dermatitis^24^. In our study, the incidence rate of these adverse events was not in contradiction to previous reports. Levy et al. also reported that the highest morbidity of acute toxicity was dermatitis (97%) with concurrent radiotherapy and cetuximab after taxane-based induction chemotherapy in locally advanced head and neck cancer^25^. Concurrent chemotherapy in NPC treated with neoadjuvant chemotherapy followed by IMRT was exhibited 48.3% dermatitis, 49.2% oral mucositis, 38.1% bone marrow toxicity, 40.9% xerostomia and 21.2% nausea vomiting^26^. There are more than 40% of patients occurred severity acute injuries for most of locoregional advanced cancer during treatment in previous studies. The investigation of IMRT dose distribution to the skin in head and neck squamous cell carcinoma was reported the incidence of grade 3 and 4 radiation dermatitis was 41.1% in patients included and 50% vs 36.6% in the cetuximab and cisplatin cohorts, respectively^27^. Grade 3–4 hematologic toxicities and radiotherapy-related oral mucositis during the period of locoregionally advanced nasopharyngeal carcinoma treated with nimotuzumab plus IMRT with chemotherapy were reported in 7.4% and 10.9% patients, and there were only 8.6% patients who complained of serious xerostomia^28^. The patients experienced severity toxic effects or cannot tolerate it during irradiation, which would either delay or suspend treatment. Nevertheless, it appears that wide rate of radiation-induced acute injuries may speculate on radiation technique, chemotherapy scheduling and the location of tumor.

Several studies have revealed that IMRT could provide reduced irradiation to the normal tissue without compromising target volume coverage, which is an advantage of the technique^29^. Data directly comparing acute side effects of NPC patients treated with IMRT and 3D-CRT are lacking in the literature as well, but lower rates of wound healing complications with IMRT were referred to more recent studies^19^. In our study, reduced hyperpigmentation and bone marrow toxicity appeared more remarkable in light of the treatment performed in the IMRT group than those of the 3D-CRT group and this difference remained robust in a univariate and multivariate analysis. As Hardee et al. demonstrated that IMRT moderately decreased rates of subacute hyperpigmentation for hypofractionated whole-breast radiotherapy at 1 month of follow-up^30^. Under this, IMRT had possessed a more homogenous dose distribution, which was reported as a good alternative to treat cervix carcinoma with bone marrow toxicity^31^. The clinical trial was demonstrated that the cause of thrombocytopenia in patients with chronic hepatitis C virus (HCV) infection, which was possibly caused by improvement of hypersplenism and HCV-induced bone marrow toxicity resulting from anti-HCV therapy^32^. Acute dermatitis and oral mucositis of the two groups were not obvious different in our study. This may have been because of strictly controlled other variables between radiation techniques. Ghosh–Laskar et al. also showed that there were no significant differences in the incidence of some acute toxicities between IMRT and 3D-CRT, such as mucositis, dermatitis and dysphagia, which is in good accordance with our findings^33^. Borm et al. released that 3D-CRT allows a homogeneous dose distribution with similar skin toxicity as compared to studies performing IMRT with chemotherapy^11^. Furthermore, a Cox proportional hazards model suggested that oral mucositis and dermatitis are truly driven by the selected IC 5-FU treated scheme or others. This can be attributed to the fact that the extra regimens of IC-CCRT may have weakened the protective effect of IMRT on the skin. However, there are some findings of previously reported studies different from above results. Liang et al. concluded that dose homogeneity across nasopharyngeal in the IMRT plan can reduce the adverse effects related to the acute skin toxicity of patients according to the RTOG toxicity criteria^34^. Katano et al. analyzed various radiation-related factors in terms of cosmetic outcome and found that the radiation techniques and doses delivered to the nasopharynx were statistically significant factors^35^. Comparing with 3D-CRT, patients treated with IMRT for anal carcinoma had a significantly lower degree of skin toxicity and higher rate of acute diarrhea, but rates of hematological toxicity and proctitis were not reduced^19^. Comparison of toxicities demonstrated that IMRT was a safe regimen with less xerostomia, acute dermatitis and favorable locoregional control, survival rates during treatment of locally advanced oropharyngeal carcinoma^36^. Since acute side affections frequently limit radiation treatment, IMRT might have enabled more aggressive treatment in some patients.

However, not all side effects of radiotherapy were superior in the IMRT group. We noted a significantly increased rate of gastrointestinal injury and leukopenia upon IMRT, observed in univariate and multivariate analyses, which is not entirely clear. One possible explanation could be a different distribution of radiation in the small intestine. This difference with higher treatment rates and dosages for 5-FU and docetaxel drugs in the IMRT group, might to some extent explain the increase in acute diarrhea in the IMRT group. There was a study also reported a trend toward more gastrointestinal toxicity in IMRT^19^. In addition, our study also demonstrated a trend toward higher hematological toxicity in the IMRT group. In contrast to that, lower hematological toxicity for IMRT compared to 3D-CRT for the treatment of cervical cancer was demonstrated in some studies. Strikingly, our multivariable analysis found that the negative impact of IMRT to leukopenia was comparable, which greater than the effect of these chemotherapy drugs on these toxicities. This finding is different from the result of Chi-square test and fully shows the superiority of Log-rank method. As shown in a simulation by Kuma et al., a cohort of cervical cancer patients receiving definitive chemoradiation was assessed that more frequent severity leukopenia was found in the IMRT group^31^. As induction and concurrent chemotherapy are the major part of the treatment, which cannot be ignored, the suggestion of this study is to minimize un-necessary irradiation dosage to decrease hematological toxicity.

Our data suggest better local tumor control upon IMRT treatment. Apparently, IMRT allows a more localized focus of radiation in the primary tumor area which might have received more radiation volume and more homogenous radiation over its entire volume for maximum oncological effects. However, previously reported studies with NPC have suggested that IMRT gives at least the same outcome in primary tumor as three-dimensional conformal techniques^22^. In addition, highly similar survival outcomes for IMRT and 3D-CRT had reported in most studies as our study showed. Yan et al. concludes that IMRT and 3D-CRT have almost the same short-term and long-term clinical effects in the treatment combined with postoperative chemotherapy of nasopharyngeal carcinoma and both of them have high effectiveness and safety^37^. No survival benefits had been observed while comparing IMRT versus 3D-CRT in cervical esophageal squamous cell carcinoma patients treated by concurrent chemoradiotherapy^38^. A National Cancer Database Analysis reports that there was no difference in OS for patients who received IMRT versus 3D-CRT, IMRT was associated with similar survival as 3D-CRT^39^. Another meta-analysis showed that the 3-year OS, DFS rates had no significant difference between the IMRT and 3D-CRT groups for gastric cancer, but IMRT might be superior to 3D-CRT in treating patients with gastric cancer in terms of local control rates^22^. But beyond that, there are also studies of IMRT patients had better tumor control than 3D-CRT with equal or better oncological results for anal carcinoma patients^19^. A meta-analysis provided the results that IC-CRT should be the most suitable regimen for LANPC in the IMRT era^40^. Improvement of survival in our study upon IC-CCRT treatment is encouraging.

Some limitations must be considered for this study. Taking a full account of the occurrence of radiation-induced acute injury is valuable. However, the grade 3 or worse of dermatitis or oral mucositis had been not investigated their deterioration time during the treatment. The severity acute injuries may better report the impact of side effects between IMRT and 3D-CRT on the treatment of patients. Nevertheless, our data provide a real-world experience on adjuvant therapy except IMRT or 3D-CRT might change outcome and side effects in radiochemotherapy for LANPC. Acute toxicity should be analyzed with caution as the follow-up of patients treated by IMRT is lower than that of 3D-RCT group. However, no difference in survival was noted between the groups. The number of tumor relapses significantly different may be explained by the difference of follow-up between the groups.

## Conclusion

In summary, our data show that IMRT can reduce local side effects of IC-CCRT scheme such as hyperpigmentation and bone marrow toxicity, but increase moist desquamation, xerostomia, gastrointestinal and hematological toxicity, enable a more aggressive radiochemotherapy. We found better tumor control rates with IMRT after the end of treatment, suggesting significant oncological benefits over 3D-CRT. The effects of acute toxicities such as anemia may lead to lower tolerance for tumor treatment, which was a factor that compromises good results. In the era of IC-CCRT, the treatment of IMRT and 3D-CRT should give sufficient consideration to the occurrence of acute injuries and take active preventive measures in time. In a combined regimen of IC followed by CCRT for the treatment of LANPC, predicting the occurrence time of various side effects is beneficial to early protection and focus on coherent treatment.

## Data Availability

The data supporting the conclusions of this article are included within the article.

## Abbreviations

LANPC: Locoregionally advanced nasopharyngeal carcinoma
RT: Radiotherapy
IC: Induction chemotherapy
CCRT: Concurrent chemoradiotherapy
IC-CCRT: Induction chemotherapy followed by concurrent chemoradiotherapy
2D: Two-dimensional
3D-CRT: Three-dimensional conformal radiotherapy
IMRT: Intensity modulated radiation therapy
KPS: Karnofsky performance score
UICC/AJCC: Union for International Cancer Control/American Joint Cancer Committee
GTV: Gross tumor volume
CTV: Clinical target volume
PTV: Planning target volume
RTOG: Radiation Therapy Oncology Group
CR: Complete remission
PR: Partial relief
OS: Overall survival
LRFS: Locoregional progression-free survival
DMFS: Distant metastasis-free survival
PFS: Progression-free survival
HR: Hazard ratio
CONSORT: The consolidated standards of reporting trials
HCV: Hepatitis C virus
