# Publisher Correction: Radiation-induced acute injury of intensity-modulated radiotherapy versus three-dimensional conformal radiotherapy in induction chemotherapy followed by concurrent chemoradiotherapy for locoregionally advanced nasopharyngeal carcinoma: a prospective cohort study

**DOI:** 10.1038/s41598-021-97814-2

**Published:** 2021-09-03

**Authors:** Zexin Yao, Bing Zhang, Jialin Huang, Lei Shi, Biao Cheng

**Affiliations:** 1 School of Public Health, Guangdong Pharmaceutical University, No. 283 Jianghai Avenue, Haizhu District, Guangzhou, 510310 China; 2 School of Pharmaceutical Sciences, Southern Medical University, Guangzhou, 510599 China

Correction to: *Scientific Reports* 10.1038/s41598-021-87170-6, published online 08 April 2021

In the original version of this Article, Lei Shi was omitted as a corresponding author. Correspondence and requests for materials should also be addressed to 964673743@qq.com.

The original Article has been corrected.

